# The Interplay Between Bone and Glucose Metabolism

**DOI:** 10.3389/fendo.2020.00122

**Published:** 2020-03-24

**Authors:** Cristiana Cipriani, Luciano Colangelo, Rachele Santori, Mario Renella, Monia Mastrantonio, Salvatore Minisola, Jessica Pepe

**Affiliations:** Department of Internal Medicine and Medical Disciplines, Sapienza University of Rome, Rome, Italy

**Keywords:** glucose, bone, metabolism, diabetes, osteoporosis, fracture

## Abstract

The multiple endocrine functions of bone other than those related to mineral metabolism, such as regulation of insulin sensitivity, glucose homeostasis, and energy metabolism, have recently been discovered. *In vitro* and murine studies investigated the impact of several molecules derived from osteoblasts and osteocytes on glucose metabolism. In addition, the effect of glucose on bone cells suggested a mutual cross-talk between bone and glucose homeostasis. In humans, these mechanisms are the pivotal determinant of the skeletal fragility associated with both type 1 and type 2 diabetes. Metabolic abnormalities associated with diabetes, such as increase in adipose tissue, reduction of lean mass, effects of hyperglycemia *per se*, production of the advanced glycation end products, diabetes-associated chronic kidney disease, and perturbation of the calcium-PTH-vitamin D metabolism, are the main mechanisms involved. Finally, there have been multiple reports of antidiabetic drugs affecting the skeleton, with differences among basic and clinical research data, as well as of anti-osteoporosis medication influencing glucose metabolism. This review focuses on the aspects linking glucose and bone metabolism by offering insight into the most recent evidence in humans.

## Introduction

Diabetes and osteoporosis are common chronic diseases with serious clinical complications. Pathophysiology of the two disorders and related complications is multifactorial, and several mechanisms are now fully recognized. In this context, research studies have actively investigated the interaction between bone and glucose metabolism. Experimental data have shown the significant detrimental effect that the perturbation of glucose metabolism has on the skeleton. Clinical studies confirmed these findings and contributed to the definition of the diabetes-associated bone disease. Moreover, studies on the endocrine function of the skeleton allowed the identification of mechanisms through which bone can modulate glucose homeostasis.

The paper reviews the most recent evidence on the mutual cross-talk between bone and glucose metabolism in humans.

## How Glucose Metabolism Influences Bone

Type 2 diabetes mellitus (T2DM) is characterized by normal-high bone mineral density (BMD) and increased fracture risk (1, 2). Several bone-derived factors may be altered during perturbation of the glucose metabolism and are reported in Table 1. The main mechanisms involved in the pathogenesis of diabetes-associated skeletal disease are summarized in the following paragraphs.

**Table 1:** Osteokines in human diseases where glucose metabolism is alterated.

**Osteokines**	**Obesity**	**Type 2 diabetes mellitus**
Osteocalcin	↓	↓
Osteoprotegerin	=	↑
Sclerostin	↑	↑ or =
Lipocalin 2	=	↓
Periostin	↑	↑
BMP 9	↓ (in mice)	↓

*↑ Positively associated*.

*↓ Negatively associated*.

*= Neutral effect*.

*BMP, bone morphogenetic protein*.

### Hyperglycemia and Adipokines

Hyperglycemia by itself has toxic effects on the differentiation of bone marrow mesenchymal cells (MSC) into adipocytes (3). In fact, high glucose levels stimulate the non-canonical Wnt/protein kinase C pathway (4) and upregulates the peroxisome proliferator-activated receptor gamma (PPARγ), resulting in increased adipogenesis and bone loss (5). Poor glycemic control in diabetes patients could therefore suppress some of the master genes, as Runx2, involved in osteoblastogenesis (6). Moreover, the increased adipogenesis in the bone marrow has a strong negative effect on bone health (7). Human studies in overweight postmenopausal women with T2DM have demonstrated the inverse association between bone marrow adipose tissue and BMD (5).

Loss of lean mass and increase in adipose tissue are other key mechanisms involved in diabetes-associated bone disease. Adipose tissue, and particularly visceral fat, produces several adipokines with different effects on bone metabolism (8–10). In experimental and human studies, adiponectin, visfatin, and omentin-1 had negative effects on bone, while leptin exerted positive actions (8–10). Additionally, the role of a specific adipo-myokine, irisin, has been recently described (11, 12). It is thought that irisin plays a positive effect on hyperglycemia by stimulating glucose uptake in muscle cells (13). As far as bone metabolism, irisin may promote osteogenic differentiation, increase in cortical bone mass and strength, and reduction in the number of osteoclasts (14). Irisin resistance has been postulated in diabetes, with a consequent theoretical loss of all these positive effects on bone mass and strength (12, 15).

Several studies have reported the association between metabolic syndrome and fragility fractures (16). Conditions that characterize the metabolic syndrome indeed induce perturbation in adipokines and cytokines secretion (9). These mechanisms, in association with the altered insulin signaling, might have an influence on bone metabolism, as documented by the reduction of bone formation markers (9). Components of the metabolic syndrome other than hyperglycemia and increased adipose tissue have been associated with poor skeletal health, as well. Increases in serum triglycerides levels have been negatively associated with BMD, particularly at the femoral neck, in postmenopausal women, and with increase in bone marrow fat in young men and women (17, 18). Finally, data have shown that arterial hypertension is associated with low BMD, mostly in relation to increases in urinary calcium excretion (19, 20).

### Advanced Glycation End-Products

Hyperglycemia may act through non-enzymatic pathways and induce the formation of advanced glycation end-products (AGEs). AGEs have a detrimental effect on the skeleton, affecting the extracellular matrix and the vessels. Additionally, *in vitro* data demonstrated that high glucose levels and AGEs increase osteocytes expression of sclerostin, a negative regulator of bone formation (21). Pre-clinical observations were confirmed by clinical studies showing that sclerostin levels are higher in pre-diabetes subjects than in controls, and correlate with insulin resistance (22).

AGEs have a negative effect on bone quality, an aspect that is undetectable by dual X-ray absorptiometry. The increase in fracture risk in T2DM is indeed observed in the setting of normal BMD. Possible explanations for such a “paradox of BMD” are the high frequency of obesity in these patients, and the well-known positive association between high BMI and high BMD (23). Additionally, the role of insulin resistance, and consequent high insulin levels, has been postulated, even though some studies failed to find a positive association with BMD independently of BMI (24). On the contrary, altered bone quality has been demonstrated in patients with T2DM, as demonstrated by studies using the high-resolution peripheral quantitative computed tomography (HR-pQCT) (25). In particular, lower cortical volumetric BMD, thickness and cross-sectional area, and higher cortical porosity were observed, defining the concept of “relative deficit” at the cortical level as characteristic of the diabetic bone disease (25).

As hyperglycemia, AGEs, and microangiopathy are thought to exert negative effects on bone health, future research will define whether and how these mechanisms may be implicated in the deterioration of the cortical bone (25).

### Systemic Mechanisms

There are several systemic mechanisms, whose stimulation is driven by altered glucose metabolism that may eventually affect bone metabolism.

Chronic kidney disease (CKD) is a common complication in diabetic patients. The intricacy between CKD and bone configures a specific metabolic disorder, the CKD-Mineral Bone Disorder (CKD-MBD), that plays an important role in the skeletal fragility associated with diabetes (26).

Disarrangement in the calcium-vitamin D-PTH axis contributes to bone loss in patients with diabetes. Poor glycemic control correlates with excessive urinary calcium loss, with subsequent stimulation of chronic PTH secretion and deleterious effects on the skeleton (27). Improvement in glucose control is associated with normalization of urinary calcium excretion (28) and may avoid stimulation of PTH secretion, with positive effects on BMD (29).

## Bone Modulation of Glucose Metabolism

Osteokines are bone-derived factors that may modulate glucose homeostasis, as demonstrated in murine models. In particular, osteocalcin (OC), bone morphogenetic protein (BMP), and sclerostin (SOST) actively participate in energy metabolism, appetite, and browning of adipose tissue (30). Few experimental studies showed the possible involvement of the receptor activator of nuclear factor-kappaB ligand (RANKL), osteoprotegerin (OPG), lipocalin-2 (LCN2), and periostin in these pathways (30, 31). However, a small number of studies have been conducted to assess how these mechanisms could interplay in the modulation of glucose metabolism by the skeleton in patients with osteoporosis and/or diabetes. The major evidence is summarized in Table 2.

**Table 2:** Effects of osteokines on glucose metabolism in humans.

**Osteokines**	**Insulin**	**Fasting glucose**	**HbA1c**	**Index of insulin resistance**
Osteocalcin	↑ β cells stimulation	↓	↓	↓
Osteoprotegerin	↓ (*in vitro*)	↑	↑	↑
Sclerostin	↓	↓	↓	↓
Lipocalin 2	↑	↑	↑	↑ or =
Periostin	↑ β cells stimulation	↑	↑	↑
BMPs	↑ β cells stimulation (BMP7)	↓ (BMP 9)	↓ (BMP 9)	↓ (BMP 9)

*↑ Positively associated*.

*↓ Negatively associated*.

*= Neutral effect*.

*HbA1c, glycated hemoglobin; BMPs, bone morphogenic proteins*.

### Osteocalcin

Osteocalcin is the most abundant osteoblast-specific, non-collagenous protein, and a key determinant of bone formation. The latest review on OC reported its role in glucose metabolism and adaptation to exercise, neuronal development, and male fertility (32) (Table 2). While this is true in mice, less evidence is available in humans. Observational studies reported that OC positively correlates with insulin sensitivity in T2DM patients and that high OC levels were associated with reduced risk of developing T2DM; other studies reported lower OC levels in diabetes and no association with the risk of T2DM (33, 34). Circulating OC comprises both the undercarboxylated (u) (the form that in mice influences glucose metabolism) and the carboxylated form. In clinical studies, the total level of OC is usually reported. Hence, such conflicting results may be ascribed to measurement bias in humans. In this context, interesting data were reported by Linossier et al. who assessed the effect of the acute increase in bone resorption on glucose metabolism in 12 healthy men exposed to microgravity (35). Authors observed that the onset of insulin-resistance was in response to increased bone resorption and concomitant to the increase of uOC (35).

### Osteoprotegerin

Osteoprotegerin is a negative regulator of bone resorption through decreasing osteoclasts development. *In vitro*, OPG treatment of pancreatic ß cell lines decreased insulin release following glucose stimulation, thus preventing exhaustion of ß cells function (36, 37). Studies in postmenopausal women with normal and impaired fasting glucose levels showed that OPG levels are positively associated with insulin resistance index (38, 39). Additionally, OPG levels are higher in pre-diabetic and diabetic adults compared to those with normal glucose tolerance (40). Finally, Daniele et al. showed that OPG levels inversely correlate with the rate of insulin-mediated total body glucose disposal, while positively correlating with fasting endogenous glucose production and hepatic insulin resistance indexes (22).

### RANKL

The RANKL is a well-known primary mediator of osteoclasts differentiation, but also regulates glucose metabolism. The inhibition of the RANKL signaling has been suggested to improve hepatic insulin sensitivity and to have a role in ß cells replication in mouse models (41). In patients with osteoporosis, inhibition of RANKL improves muscle strength and insulin sensitivity (42).

### FGF23

FGF23, the key regulator of phosphate metabolism, has also been recently associated with fat metabolism (43). Data in 1,179 middle aged subjects showed that FGF23 levels were positively and independently associated with visceral obesity (44). Conversely, significant negative correlations between FGF23 levels and both fasting insulin and C-peptide levels were described in obese children and adolescents with hepatic steatosis (45).

### Sclerostin

Slerostin is a well-known inhibitor of osteoblast differentiation acting through the Wnt signaling pathway. This pathway is also active in organs involved in glucose homeostasis, such as pancreas, adipose tissue, liver, and skeletal muscle (46). Clinical data showed negative correlations between sclerostin, insulin, and homeostasis model assessment of insulin resistance (HOMA-IR) in 55 children and adolescents with simple obesity (47). In a small cohort of girls with type 1 diabetes (T1DM), a negative association between serum sclerostin levels and glycated hemoglobin (HbA1c) was found (48). As far as adult patients, studies have shown that those with T1DM had either higher or comparable values of sclerostin compared to controls, while an increase in sclerostin levels is described in patients with T2DM (49–51). Finally, results from the Canadian Multicentre Osteoporosis Study (CaMos) showed that sclerostin levels are associated with fasting insulin levels and HOMA-IR, but not with the risk of incident T2DM (52).

### Lipocalin-2

Lipocalin-2 has a prominent role in the pathological response of bone tissue to low mechanical forces in murine models (53). Recently, data in mice have shown that LCN2 crosses the blood-brain barrier and binds to specific neurons of the hypothalamus to control appetite (54). In humans, ROC curve analyses demonstrated that LCN-2 levels could discriminate between normal subjects and those with impaired glucose tolerance (IGT), as well as T2DM and IGT among obese women (55). In particular, significant positive correlation between LCN2 levels and fasting glucose and 2-h postprandial blood glucose, serum insulin, HbA1c, HOMA-IR was detected (55–57).

### Periostin

Periostin is a matricellular protein derived from osteoblast and osteocytes. In mice, periostin is able to potentiate pancreatic β-cell regeneration, and is involved in the inhibition of sclerostin following skeletal mechanical loading (58, 59). In humans, high plasma periostin levels were observed in a study assessing 161 obese Chinese patients with T2DM (60). Periostin was strongly associated with triglyceride metabolism, chronic inflammation, and insulin resistance (60). In a cross-sectional study of 8,850 subjects aged 40 or older, periostin positively correlated with liver function, triglycerides levels, waist circumference, HOMA-IR, and fasting plasma insulin in overweight and obese subjects (61).

### Bone Morphogenetic Proteins

Bone morphogenetic proteins control both osteoblasts and osteoclasts' function. These molecules are released from the bone matrix into circulation during bone resorption, thus may have effects on organs apart from the skeleton (30). Receptors of the BMPs were indeed found in multiple organs, such as the liver (62). Hence, regulation of bone turnover by BMPs may be coupled with their effect on organs related to glucose metabolism. Interestingly, *in vitro* data have reported the conversion of primary human pancreatic exocrine tissue into functional islet endocrine cells after exposure to BMP-7 (63).

In humans, circulating BMP-9 levels were found to be significantly higher in healthy subjects than in newly diagnosed T2DM patients and negatively correlated with HbA1c, fasting glucose, and HOMA-IR (64).

## Anti-Diabetic Drugs That Interfere With Bone Metabolism

Anti-diabetic drugs may have detrimental, positive, and neutral effects on bone metabolism. In this context, experimental studies are often not corroborated by clinical data.

### Metformin and Sulfonylureas

Metformin and sulfonylureas have no clinical significant effect on bone in humans. In pre-clinical studies, metformin activates differentiation of the mesenchymal stem cells toward the osteoblastic lineage while inhibiting adipogenesis and osteoclast differentiation (65–67). Clinical studies have shown inconsistent results on the effect of metformin on fracture risk. Some observational and retrospective studies and recent meta-analyses reported reduction in fracture occurrence in diabetic patients treated with metformin, while others did not observe any significant effect (68–72). Whether these results could be related to the overall low fracture risk of metformin users (72) or whether the use of metformin may have clinically significant protective effects on the skeleton needs to be addressed by future randomized prospective studies.

Pre-clinical data have shown the potential of sulfonylureas, particularly glimepiride, in stimulating bone formation (73, 74). Data in ovariectomized rats have shown that glimepiride could inhibit skeletal changes associated with menopause while stimulating bone formation (75). Clinical data have essentially reported a neutral effect of sulfonylureas on BMD and/or fractures (76–78). There are no clinical trials designed to assess fractures and/or falls as the primary endpoint in sulfonylureas users, in which the main fracture risk is hypoglycemia, particularly in older and frail individuals (78–80).

### Insulin

Similarly to what is discussed for sulfonylureas, clinical effects of insulin on bone are mostly driven by the occurrence of hypoglycemia and consequent fracturing (81). The anabolic effects of insulin seen in experimental studies do not indeed translate into positive effects on bone health in humans (81). A recent population-based study of 58,853 newly diagnosed diabetic patients reported a 38% excess risk of major osteoporotic fractures in those treated with insulin (82).

### Thiazolidinediones

Thiazolidinediones have been associated with reduction in BMD and increased incidence of fractures (83, 84). Rosiglitazone was associated with 6–20% increase in bone resorption and 4–13% reduction in bone formation markers in postmenopausal women, and with clinically significant bone loss (69, 83, 85–87). Similar data are available for pioglitazone (88, 89). Meta-analyses of studies have demonstrated that treatment with thiazolidinediones is associated with increased fracture risk in postmenopausal women, with a possible association to duration of therapy (73, 83). Main mechanisms include loss of the inhibitory effect of PPAR-γ on osteoclasts differentiation, increased production of sclerostin and DKK1 in osteocytes, and infiltration of adipocytes in the bone marrow (65).

### Incretins

The presence of the GLP-1 receptor on the pre-osteoblasts and osteocytes surface was associated with the anabolic actions of liraglutide and exenatide in murine models (90–92). In humans, exenatide and liraglutide treatment were found to prevent bone loss associated with weight reduction, and a 16% increase in P1NP serum levels was observed in liraglutide-treated patients (93, 94). Results on the effect of GLP-1 agonists on fracture are inconclusive (65, 95), even though a recent meta-analysis suggests that treatment with liraglutide and lisixenatide were associated with decreased fracture events (96).

### DPP4-Inhibitor

Suppression of bone resorption has been reported in association with DPP4-inhibitor sitagliptin in murine models, and only one study reported similar results in postmenopausal women (97, 98). While clinical studies showed a possible decrease in fracture risk in DPP4-inhibitors users, recent meta-analyses showed that this class of drug does not influence fracture risk when compared to placebo or other anti-diabetic agents (99–102).

### Sodium-Glucose Cotransporter-2 Inhibitors

Possibly harmful effects on the skeleton from sodium-glucose cotransporter-2 inhibitors have been observed in the first clinical trials, but not confirmed by the subsequent data. Mechanisms such as weight loss, and increased urinary calcium excretion and PTH levels have been postulated (65). Data from the CANVAS study has reported a 4% incidence of fractures that was significantly higher vs. placebo (103). There was instead no difference in the incidence of fracture in the CANVAS-R study in the canaglifozin vs. placebo group, nor in the most recent CREDENCE trial (104, 105). Finally, the analysis of data from the CANVAS and the CANVAS-R trials comprising 10,142 participants concluded that data from the CANVAS trial could be related to chance or presumably to the presence of falls, whose prevalence was not specifically recorded in all studies (106).

As far as other agents of this class, pooled analysis of data in over 12,000 patients from placebo-controlled and head-to-head trials vs. glimepiride excluded the association of empaglifozin with fractures (77).

## Anti-Osteoporosis Drugs That Interfere With Glucose Metabolism

Drugs currently used for treatment of osteoporosis modify bone turnover markers, which in turn could modify glucose metabolism. Reduction in bone turnover markers is observed during bisphosphonates and denosumab treatment (antiresorptive agents), while enhanced bone turnover is observed during PTH 1-34 and PTH 1-84 administration (anabolic agents).

### Bisphosphonates

Registrative studies of alendronate, zoledronic acid, and denosumab did not report any significant effects of these drugs on incident diabetes or fasting glucose in postmenopausal women (107). Conversely, a retrospective population-based study conducted in Taiwan showed that the use of alendronate decreases the incidence of diabetes in subjects younger than 65 without dyslipidemia and hypertension (108). Reduction in the risk of diabetes was observed in another population-based retrospective study conducted in UK in individuals aged 60 and older with no baseline diabetes and more than 1 year of bisphosphonates exposure (109).

A recent randomized controlled trial (RCT) showed a significant decrease of 8.2 mg/dL in fasting glucose levels and of 0.2% in HbA1c levels in postmenopausal women with osteopenia and pre-diabetes treated with alendronate (110).

### Denosumab

A *post-hoc* analysis of the FREEDOM trial showed no effect of denosumab on fasting glucose levels in postmenopausal women with diabetes and pre-diabetes. A modest decrease in fasting serum glucose (−6.8 mg/dL) was observed only in women with diabetes not using antidiabetic medications (111). A prospective study by Passeri et al. reported that a single dose of 60 mg of denosumab was not associated with changes in the glucose or insulin response to OGTT in non-diabetic postmenopausal women with osteoporosis (112). Authors observed a modest significant reduction in hepatic insulin resistance index only at 4 weeks (112). Similar results were observed in a subsequent study assessing the glucometabolic parameters and lipid profile in 48 non-diabetic osteoporotic postmenopausal women at 24 weeks after a single 60 mg denosumab dose (113). No significant changes were observed, with the exception of a significant reduction in insulin and HOMA-IR at 4 weeks (113).

### Anabolic Agents

Anastasilakis et al. have shown that the intermittent administration of PTH 1-34 is followed by a subtle transient increase in calcium and PTH levels with no effect on glucose homeostasis (114).

As far as PTH 1-84 is concerned, a randomized, controlled, open-label trial involving 46 postmenopausal non-diabetic women with osteoporosis has shown that the hormone increases both OC and uOC, and decreases fasting plasma glucose (115). Interestingly, the effects of PTH 1-84 on OC and on uOC represented the mediator of more than the half (62%) at 12 months and almost half (48%) at 6 months, respectively, of the total effect of these hormones on fasting glucose (115).

### Vitamin D and Calcium

Data from clinical studies showed that calcium and vitamin D supplementation might exert beneficial effect on glucose metabolism. However, different results have been observed among studies (116, 117). The most recent meta-analysis including 12 studies with 4,395 participants in the intervention arm and 4,551 in the control group demonstrated that calcium and vitamin D supplementation significantly reduce fasting glucose, HOMA-IR, and insulin levels (116).

## Conclusions

Bone and glucose metabolism are strongly interrelated. Experimental studies have assessed the mechanisms of the mutual cross-talk between bone and glucose homeostasis, and allowed for a definition of diabetes-associated bone disease, for which a more proactive clinical evaluation and treatment is now recommended (118). Moreover, data on the endocrine actions of bone on glucose and energy metabolism have opened new and interesting insight into the possible risk of diabetes and obesity in patients with metabolic bone disease.

Future studies, particularly RCTs, will define the effect of anti-osteoporosis medications on glucose homeostasis, as well as how anti-diabetic agents could impact bone health.

## Author Contributions

CC and JP contributed to ideation, drafting, and revising of the manuscript. LC, RS, MR, and MM contributed to the literature search and drafting of the manuscript. SM contributed to ideation and revising of the manuscript. All authors have revised and accepted the final version of the manuscript.

### Conflict of Interest

The authors declare that the research was conducted in the absence of any commercial or financial relationships that could be construed as a potential conflict of interest.
